# Precision Exercise for Breast Cancer-Related Outcomes: Towards Personalised Training Based on Tumour, Treatment and Patient Characteristics

**DOI:** 10.1007/s40279-026-02481-w

**Published:** 2026-06-30

**Authors:** Cristina Crespo-Garcia, Anita Borsati, Dennis R. Taaffe, Carolyn J. Peddle-McIntyre, John P. Campbell, Alice Avancini, Emily Jeffery, Daniel A. Galvao, Robert U. Newton

**Affiliations:** 1 Exercise Medicine Research Institute, Edith Cowan University, 270 Joondalup Drive, Joondalup, Perth, WA 6027 Australia; 2 School of Medical and Health Sciences, Edith Cowan University, Perth, WA Australia; 3 Biomedical, Clinical and Experimental Sciences, Department of Medicine, University of Verona, Verona, Italy; 4 Department of Neurosciences, Biomedicine and Movement, University of Verona, Verona, Italy; 5 Department for Health, University of Bath, Bath, UK; 6 Section of Innovation Biomedicine-Oncology Area, Department of Engineering for Innovation Medicine (DIMI), University of Verona and University and Hospital Trust (AOUI) of Verona, Verona, Italy; 7 Curtin School of Population Health, Curtin University, Perth, WA Australia; 8 School of Human Movement and Nutrition Sciences, University of Queensland, Brisbane, QLD Australia

## Abstract

Exercise is a promising strategy associated with improved cancer-related outcomes through multiple biological mechanisms. However, current exercise guidelines largely adopt a one-size-fits-all approach and overlook inter-individual variability in response. Given the heterogeneity of breast cancer, precision exercise prescriptions may better target biological pathways linked to therapeutic efficacy and long-term prognosis. Observational studies consistently show that higher physical activity levels are associated with improved survival outcomes, including reduced all-cause and breast cancer-specific mortality. In contrast, evidence from interventional trials remains limited and inconsistent, with effects on tumour response and survival largely derived from secondary or exploratory analyses. These discrepancies likely reflect differences in study design, exercise dose, adherence and treatment-related factors, and may indicate that null findings are partly attributable to an insufficient or inconsistent exercise stimulus rather than a true biological non-response. Exercise influences key pathways involved in tumour progression and treatment response, including immune function, inflammation, metabolism, tumour perfusion and the gut microbiome. As these responses may vary according to tumour subtype, treatment context and host characteristics, a uniform approach to exercise prescription may fail to optimise its therapeutic potential. This review proposes a precision exercise framework that integrates tumour, treatment and patient-specific factors such as genetic and epigenetic profiles, metabolic status and body composition, systemic inflammation and the gut microbiome to guide precision exercise strategies. It also highlights the potential of digital tools, including wearable technologies and artificial intelligence, to enable data-driven personalisation and real-time adaptation, alongside key considerations for safe clinical implementation. While this framework is supported by biological plausibility, whether variability in these upstream biological responses translates into differences in cancer-related outcomes remains unknown. As such, this approach remains hypothesis generating. Future research should focus on adequately powered trials and mechanistic studies to determine whether precision exercise can meaningfully improve cancer-related outcomes in breast cancer.

## Key Points

**Table:** 

Precision exercise, tailored to tumour subtype, treatment context and host-specific factors (e.g. genetics, inflammation, microbiome), may help target biological mechanisms relevant to improve therapeutic efficacy and survival in patients with breast cancer.
Emerging evidence supports that different exercise modalities influence immune, metabolic and molecular pathways differently depending on individual characteristics.
Integrating clinical, biological and digital data through omics, wearables and artificial intelligence holds promise for transitioning from a one-size-fits-all model to precision exercise oncology.

## Introduction

Precision exercise medicine in the oncology setting refers to the tailoring of exercise prescriptions based on a patient’s clinical, physiological and tumour-specific characteristics to optimise clinical outcomes in patients with cancer [1, 2]. Exercise is associated with reduced cancer risk and improved prognosis, with several biological mechanisms having been proposed, including reduced sex hormone levels, decreased inflammation, improved insulin sensitivity, favourable body composition, release of anti-proliferative myokines and enhanced immune function [3–7].

Exercise can be considered to consist of distinct types of “medicines,” including aerobic, resistance, flexibility and balance training. Key exercise characteristics (frequency, intensity, and time) and principles of training (such as specificity, progressive overload, autoregulation and periodisation) can be strategically manipulated to target specific objectives [1, 8]. However, the prescription and delivery of exercise are poorly reported across exercise medicine, which limits replication and translation to clinical practice [9]. In oncology, exercise guidelines largely adopt a one-size-fits-all model and rarely provide detailed prescriptions for training variables or stratification by cancer type, stage, tumour-specific biological features or co-morbidities [10, 11]. Given that, in many patients, morbidity and mortality are driven not only by cancer progression but also by treatment-related toxicities, comorbid conditions and functional decline, including cardiovascular disease, sarcopenia and frailty, accounting for these factors is essential in clinical exercise prescription. Accordingly, some frameworks now address comorbidities and treatment-related side effects, such as cardiovascular disease, frailty, fatigue, sarcopenia or lymphedema. However, few address how exercise could be tailored to enhance treatment efficacy, reduce recurrence risk or target key biological pathways, including immune function, inflammation, hormonal regulation, insulin sensitivity or the gut microbiome [10, 11].

Importantly, not all individuals respond equally to exercise, highlighting the need for individualised approaches [1]. Evidence from both healthy and clinical populations demonstrates substantial variability in short-term physiological responses and longer-term phenotypic adaptations to exercise, such as changes in cardiorespiratory fitness, muscular strength, body composition or metabolic markers, supporting the classification of individuals as non-, low or high responders [12–14], However, responder status is outcome specific and depends on the physiological or biological endpoint considered [15, 16]. These heterogeneous responses arise from both biological (e.g. genetic, hormonal, metabolic status) and contextual (e.g. lifestyle, comorbidities, treatment) factors [13, 16]. Nevertheless, a limited observable effect in some individuals may not necessarily reflect biological resistance to exercise but may also reflect an insufficient or inappropriate training stimulus [17]. In healthy populations, appropriate adjustments in intensity, volume or modality, enable meaningful physiological adaptations across individuals, suggesting that a similar precision exercise approach may be relevant to explore in clinical populations [13, 16, 17].

Importantly, most evidence for inter-individual variability relates to physiological and patient-reported outcomes. However, whether this variability translates to cancer-related outcomes (e.g. tumour response, and survival outcomes such as recurrence and survival) remains largely unknown. The rationale for extending this concept to cancer-related outcomes is therefore based on biological plausibility. Exercise influences key pathways involved in tumour progression and treatment response, including immune function, inflammation, metabolism and tumour perfusion. Variability in these upstream responses may contribute to heterogeneity in downstream cancer-related outcomes, although this remains to be confirmed in clinical studies.

**Fig. 1:**
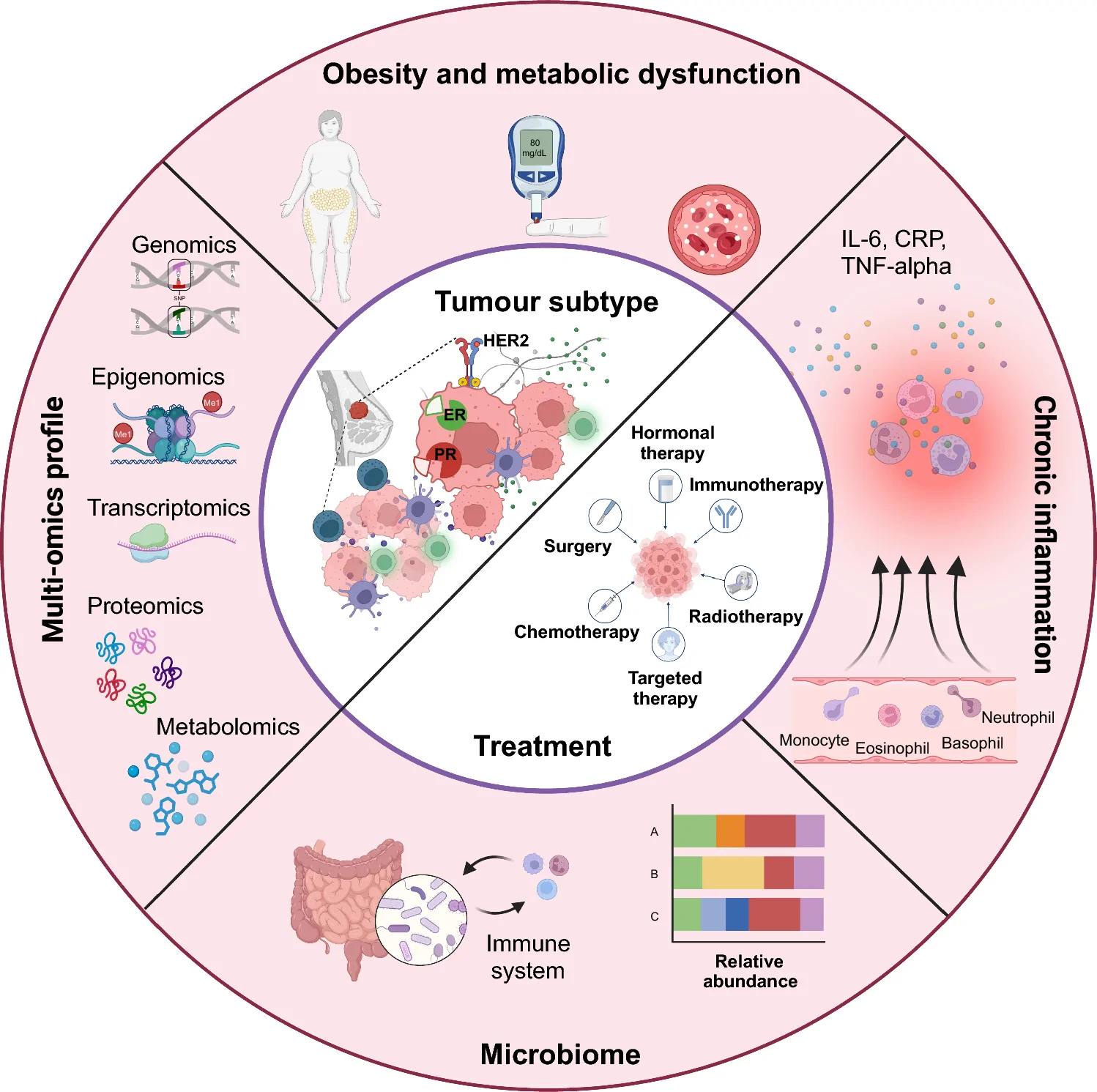
Conceptual model of key factors informing precision exercise prescription in breast cancer. The inner ring reflects tumour-specific characteristics, including subtype and treatment context, which may inform the development of precision exercise interventions. The outer ring illustrates patient-specific factors, such as the multi-omics profile, obesity and metabolic dysfunction, gut microbiome and chronic inflammation, which may either influence how patients respond to exercise or help identify biological processes to target (e.g. reducing inflammation or improving metabolic health). This model is conceptual and intends to guide future research and hypothesis generation in precision exercise oncology. *CRP* C-reactive protein *ER* estrogen receptor, *HER2* human epidermal growth factor receptor 2 positive, *IL-6* interleukin 6, *PR* progesterone receptor, *TNF*-alpha tumour necrosis factor alpha

**Fig. 2:**
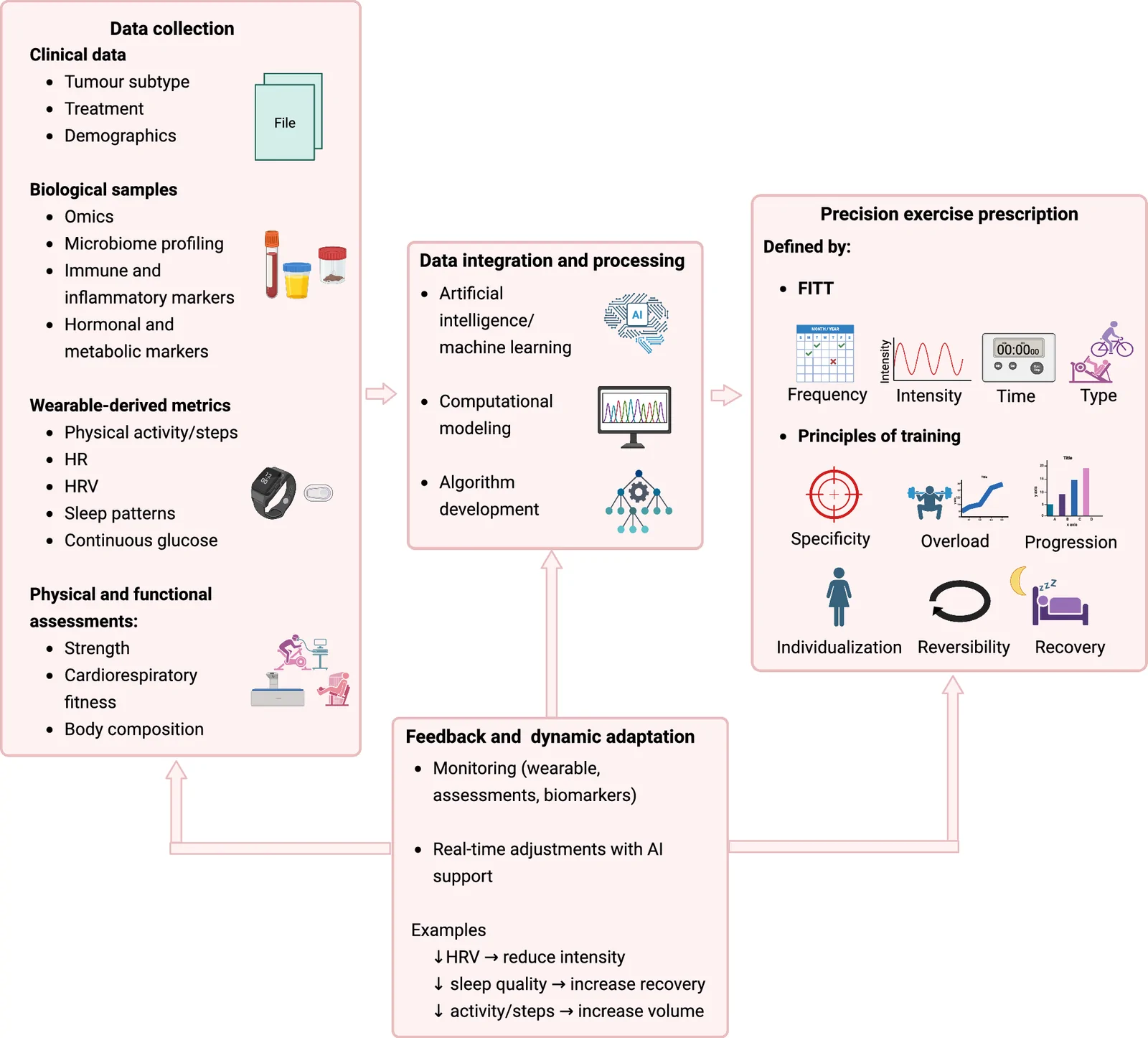
Proposed approach for developing precision exercise programmes in breast cancer. Clinical data (e.g. tumour subtype, treatment regime, patient demographics), biological samples (e.g. blood, tumour tissue, multi-omics), physical and functional assessments, and wearable device outputs are collected to generate an integrated dataset. These data are processed using advanced analytical tools such as machine learning, computational modelling and algorithm development. These data may ultimately inform the design of precision exercise prescriptions tailored to tumour subtype, treatment context and patient-specific characteristics, applying FITT (frequency, intensity, time, type) principles and core training concepts. While this approach remains conceptual and exploratory, it highlights the potential pathway toward implementing biologically informed, precision exercise strategies to improve clinical outcomes in breast cancer. *AI* artificial intelligence, *HR* heart rate, *HRV* heart rate variability, ↓ decreased

To improve therapeutic precision, future exercise strategies should be matched to both tumour biology and host-specific characteristics, such as genetic and epigenetic profiles, microbiome characteristics, inflammatory status and treatment type [1, 2, 6, 10] (Fig. 1). By integrating molecular data and clinical features, exercise could be prescribed with the same precision intent as medical therapies (Fig. 2).

This narrative review explores current evidence on physical activity and exercise in relation to breast cancer prognosis, with a focus on tailoring interventions to tumour, treatment and patient-specific characteristics. Physical activity is defined as any bodily movement produced by skeletal muscles that results in energy expenditure, whereas exercise is a subset of physical activity that is planned, structured and repetitive, with the objective of improving or maintaining physical fitness [18]. First, we summarise findings from observational and interventional studies on post-diagnosis physical activity and exercise in relation to survival outcomes and tumour response. We then examine the rationale and emerging evidence supporting a precision exercise approach, including how exercise may be adapted based on tumour subtype, treatment and individual characteristics. Next, we address key considerations for safe and effective clinical implementation, including treatment-related toxicities, contraindications, and the need for individualised progression and monitoring. Finally, we discuss emerging tools, such as omics technologies, wearables and artificial intelligence (AI), that may support data-driven personalisation and highlight key research gaps. Given the current limitations in clinical evidence, this framework should be considered hypothesis generating rather than conclusive.

## Methods

This article is a narrative review synthesising current evidence on precision exercise in breast cancer, with emphasis on tumour biology, treatment context and patient-specific characteristics. A literature search was conducted in PubMed/MEDLINE complemented by manual screening of reference lists from key reviews and influential studies. Searches were conducted between January and June 2025, using combinations of terms related to precision exercise, breast cancer, survival, tumour response, exercise or physical activity, tumour subtype, chemotherapy, radiotherapy, immunotherapy, immune response, metabolism, obesity, inflammation, genetics, epigenetics and gut microbiome.

Eligible publications included peer-reviewed original research articles, randomised controlled trials (RCTs), prospective cohort studies, systematic reviews and meta-analyses published in English. Studies focusing on breast cancer populations were prioritised; evidence from mixed tumour cohorts or preclinical models was included when providing relevant mechanistic insight and when clinical breast cancer data were limited.

Articles were initially screened by title and abstract, followed by a full-text assessment. Evidence was synthesised qualitatively, prioritising meta-analyses and RCTs, followed by key observational and mechanistic studies, to identify patterns, heterogeneity and knowledge gaps relevant to precision exercise approaches.

## Physical Activity and Breast Cancer Prognosis: Data from Observational Studies

Physical activity (primarily referring to habitual or self‑reported movement behaviours) has been extensively studied in relation to survival outcomes, such as all-cause mortality, cancer-specific mortality and recurrence. A recent meta-analysis of 20 cohort studies involving 31,563 women found a significant inverse association between higher levels of post-diagnosis recreational physical activity and both all-cause mortality (48% lower risk) and breast cancer-specific mortality (38% lower risk) [19]. Additionally, a linear dose–response relationship was observed: for every 10 metabolic equivalent of task (MET)-hours per week of physical activity, the risk of all-cause and breast cancer-specific mortality decreased by 15% and 14%, respectively, independent of potential confounders such as menopausal status, body mass index (BMI), tumour molecular subtype and physical activity intensity. The strongest benefits have been observed at 15–20 MET-hours/week of activity, equivalent to 150–300 min/week of moderate-to-vigorous activity, beyond which no additional improvements were seen (non-linear dose–response) [19, 20]. For risk of recurrence, no association was found between recreational physical activity and breast cancer recurrence; however, the number of included studies was smaller [19].

At the pooled level, the meta-analysis by Cariolou et al*.* [19] reported improved survival across all hormone receptor subtypes, suggesting no major differences by tumour biology. However, early observational studies have reported stronger survival benefits in hormone receptor-positive (HR+) tumours [10, 21], suggesting potential heterogeneity in response and supporting the concept of precision exercise oncology. More recently, data from the Nurses’ Health Studies reported reduced all-cause mortality with higher activity levels, but only in estrogen receptor-positive (ER)+/progesterone receptor-positive patients [22], while Soldato et al. [23] found that post-diagnosis physical activity (measured before the start of treatment) was associated with reduced distant recurrence only in women with triple-negative breast cancer (TNBC) and human epidermal growth factor receptor-positive (HER2)+ subtypes, especially among premenopausal women. These discrepancies likely reflect differences in cohort characteristics, timing of physical activity level assessment, treatment advances, and analytic methods, underscoring the need for more research to clarify whether and how tumour subtype may influence exercise responsiveness.

## Exercise and Breast Cancer-Related Outcomes: Data from Interventional Studies

Few RCTs have evaluated the effects of exercise interventions (defined as planned, structured, and prescribed training programmes) on survival outcomes, including overall survival (OS) and disease-free survival (DFS) in breast cancer, with existing evidence originating from exploratory follow-up analyses, limiting causal interpretation.The Supervised Trial of Aerobic versus Resistance Training (START) trial by Courneya et al. [24] was a multicentre trial that randomised 242 patients with breast cancer to usual care, supervised aerobic exercise training or resistance exercise training during adjuvant chemotherapy (median of 17 weeks). In an exploratory analysis after a follow-up period of 8 years, women randomised to the exercise groups combined showed a non-statistically significant improvement in DFS (82.7% vs 75.6%) and OS (91.2% vs 82.7%) compared with usual care [24]. Notably, subgroup analyses reported stronger effects among women with ER+ and HER2+ tumours for DFS and recurrence-free interval. Similarly, exploratory analyses in the Exercise for Health trials [25] which delivered an 8-month unsupervised combined aerobic and resistance training intervention starting 6 weeks post-surgery, reported significantly higher OS (hazard ratio = 0.44, 95% confidence interval [CI] 0.19–0.98, *p* = 0.046) and DFS in the intervention group after a median 8.3-year follow-up (20).

In stage III colon cancer, a recent RCT showed that a 3-year structured exercise programme post-chemotherapy improved DFS (hazard ratio = 0.74), with findings also suggesting longer OS [26]. Although not in breast cancer, this is the first RCT to demonstrate survival benefits from exercise in solid tumours, supporting the need for breast cancer-specific RCTs with survival endpoints; however, extrapolation to breast cancer should be made cautiously.

Beyond survival, few studies have explored whether exercise during neoadjuvant chemotherapy (NACT) can improve tumour response (e.g. pathologic response), with findings remaining inconsistent across trials and largely exploratory. The Lifestyle, Exercise, and Nutrition Early After Diagnosis (LEANer) RCT by Sanft et al. [27] remains the only trial to show a significant benefit. In this study, 173 women with breast cancer received a home-based intervention combining aerobic and resistance training with nutritional counselling during NACT, aiming for ≥ 150 min per week of moderate-to-vigorous physical activity and twice-weekly sessions of strength training. The intervention group had significantly higher pathologic complete response (pCR) rates (53% vs 28%, *p* = 0.037), with exploratory analyses suggesting that this effect may be restricted to HR+/HER2− and TNBC subtypes. In contrast, in a secondary analysis of a phase II trial, Iyengar et al. [28] found no differences in overall pCR rates following supervised aerobic training consisting of individualised treadmill walking three times per week (20–50 min per session) at 55–100% of pre-treatment peak oxygen consumption. Importantly, although overall pCR rates did not differ, exploratory analyses suggested a potential benefit in patients with HR+/HER2− tumours. Similarly, the recent BENEFIT RCT [29] reported no improvements in tumour size or pCR rates across groups following supervised aerobic or resistance training during NACT in 180 patients with breast cancer. The 12-week programme included twice-weekly sessions of either resistance training (60–80% of the participant’s 1-repetition maximum) or aerobic training (continuous moderate intensity at 60–70% of maximal oxygen consumption (VO_2_max) in the first 6 weeks, followed by high-intensity intervals from week 7 onwards). Nevertheless, exploratory subgroup analyses suggested a potential signal in HR+ patients, whereby aerobic training was associated with higher odds of tumour shrinkage (odds ratio = 2.37, 95% CI 0.97–5.78) and pCR (odds ratio = 3.21, 95% CI 0.97–10.61) compared with usual care [29]. Both aerobic and resistance training also appeared to reduce the need for axillary lymph node dissection in HR+ patients, although not significantly. Notably, session attendance declined over the course of NACT, possibly underestimating the true effects of exercise [29]. Other studies of supervised combined aerobic and resistance training programmes during NACT have reported null findings. Guedes et al. [30] found no significant differences in pCR rates and Rao et al*.* [31] observed only a non-significant reduction in tumour proliferation (Ki-67), but no differences in tumour size or nodal status, in overweight women with ER+ tumours.

The overall evidence base remains limited and heterogeneous and remains insufficient to establish a causal effect of exercise on survival outcomes or tumour response in breast cancer. Several key limitations should be acknowledged. First, most evidence is from observational studies, which cannot establish causality. A further concern is the reliance on self-reported physical activity data, often collected through questionnaires, which may lead to over- or under-estimation of activity levels [32, 33]. Moreover, most current RCTs were not designed with survival or tumour response as primary outcomes and reported effects on these outcomes are largely derived from secondary or exploratory analyses. In addition, trials vary widely in design, exercise modality, exercise dosage and outcome measures, making it difficult to identify the most effective type and dose of exercise, which may be further complicated in home-based interventions. Finally, not all patients derive equal benefit from physical activity. Exploratory subgroup analyses suggest greater effects in younger women and those with stage II + disease [25] and in HR+/HER2− [28] and HR+ subtypes [29], although this remains to be confirmed.

### Modifiers of Exercise Effects

Beyond the general limitations mentioned above, issues related to the delivery of exercise interventions are also likely to have influenced observed outcomes. Exercise programmes are frequently delivered at a suboptimal intensity or dose [34], while variability in adherence, supervision and progression may substantially influence the effective training stimulus received. For example, in the study by Iyengar et al. [28], median exercise adherence was approximately 77%, suggesting that a considerable proportion of participants did not complete the prescribed training stimulus.

Treatment-related factors further complicate intervention delivery. A high prevalence of symptoms such as fatigue and nausea, reported in up to 80% of patients undergoing chemotherapy [27], as well as clinically relevant toxicities including neutropenia, anaemia, peripheral neuropathy and cardiotoxicity, may limit patients’ ability to sustain adequate exercise intensity and volume, reduce adherence and compromise intervention fidelity. Similarly, in the BENEFIT trial, premature discontinuation of chemotherapy because of adverse events further reduced both intervention fidelity and effective exercise exposure [29].

Collectively, these findings suggest that null or modest effects observed in current trials may not necessarily reflect a lack of biological responsiveness to exercise, but rather an insufficient or inconsistent exercise stimulus delivered under the constraints of active cancer treatment. In this context, apparent “non-response” is often outcome specific and influenced by modifiable factors such as exercise dose, intensity, duration, modality, adherence and progression, indicating that targeted adjustments to the training stimulus may mitigate these effects rather than representing true biological resistance to exercise [15, 16]. In addition, during active cancer treatment, the goal of exercise interventions may not always be to induce improvements, but rather to attenuate or prevent treatment-related declines in physical, metabolic and immune function. In this context, the maintenance of function or a smaller deterioration may represent a clinically meaningful benefit. Together, these considerations emphasise the importance of interpreting exercise responses within the constraints of active cancer treatment.

## Precision Exercise

The following sections outline a conceptual and hypothesis-generating framework for precision exercise in breast cancer, integrating tumour, treatment and patient-specific characteristics to propose potential biological targets and exercise strategies. This framework is intended to illustrate plausible mechanisms and guide future research, rather than to provide definitive or clinically actionable recommendations.

### Tailoring Exercise Based on Tumour Subtype and Treatment

#### Tumour Subtype

Breast cancer subtypes are typically classified into four categories based on immunohistochemical expression of different receptors: ER+, progesterone receptor-positive, HER2+ and TNBC, which lacks expression of all three receptors [35]. As discussed earlier, emerging evidence suggests potential variability in exercise responses across tumour subtypes. This may be explained by underlying biological differences between subtypes, including variations in hormonal signalling, immune infiltration and metabolic pathways, which could influence responsiveness to exercise.

##### Hormone-Positive Tumours

Mechanistically, ER+ tumours, which rely on circulating estrogens for growth, may benefit from exercise through reductions in circulating estrogens and increases in sex hormone-binding globulin [36, 37]. Recent meta-analyses have shown that physical activity and exercise lead to favourable changes in sex hormone profiles, including increased sex hormone-binding globulin and decreased levels of estrogens, androgens and progesterone [38, 39]. Both aerobic and resistance exercise produce beneficial effects, with no clear superiority of one modality over the other [39]. These hormonal changes have been observed regardless of menopausal status, though stronger responses are typically reported in overweight women and when weight loss occurs [39]. Notably, reductions in total oestradiol are related to weight loss, whereas decreases in free oestradiol occur independently of weight loss, likely reflecting increased sequestration of circulating oestradiol by sex hormone-binding globulin [38].

An additional mechanism through which exercise may reduce estrogen availability is by reducing adiposity and lowering systemic inflammation, which in turn decrease aromatase activity, the enzyme responsible for converting androgens into estrogens, thereby lowering estrogen production [36, 40, 41]. In this context, multimodal dietary and combined aerobic and resistance training interventions that improve metabolic health and reduce adiposity and inflammation, with resistance training preventing muscle loss, may offer added benefit for patients with HR+ breast cancer [36, 42].

##### HER2+ and TNBC

Human epidermal growth factor receptor-positive tumours, and especially TNBC, are more immunogenic than HR+/HER2− tumours and have higher levels of tumour-infiltrating lymphocytes and programmed death-ligand 1 expression [43, 44]. Additionally, tumour mutational burden, a measure of the number of somatic mutations per megabase, tends to be higher in TNBC than in HR+ or HER2+ tumours [45]. These features suggest greater potential for immune-mediated antitumour effects of exercise in these subtypes [46, 47], including enhanced immune infiltration, increased naïve T-cell output, reduced dysfunctional T cells and reprogramming of the tumour microenvironment [47–49]. Preclinical studies in mice confirm that treadmill and wheel running can promote anti-tumour immunity and reduce tumour growth in HER2+ and TNBC types [50–56].

Exercise-induced release of myokines, particularly interleukin (IL)-6, IL-7, and IL-15, further support antitumour immunity by sustaining naïve and memory T cells and promoting natural killer (NK) cell differentiation and infiltration into tumours [48, 57]. Studies in non-cancer populations suggest that moderate-to-high intensity exercise of at least 30 min appears necessary to elicit robust myokine responses [58]. Moreover, myokine levels typically peak within 60 min post-exercise and return to baseline within 24 h [48, 59], indicating that frequent regular training may be necessary to sustain the immune-enhancing effects.

Despite promising preclinical evidence, the effects of exercise on immune cell populations within human tumours remain poorly understood. To date, only two studies have directly assessed intra-tumoural immune changes in patients with breast cancer, with a recent pilot RCT providing preliminary evidence that exercise during NACT may favourably modulate these outcomes. In this trial, participants underwent a supervised combined aerobic and resistance training programme at moderate-to-high intensity, performed twice weekly for 6 weeks. In the exercise group, there was a higher tumour infiltration of CD56+ cells, indicating higher levels of NK cells and/or CD8^+^ T cells, whereas a decline was observed in the control group [60]. The exercise group also showed a reduction in the intra-tumoural CD4/CD8 ratio, suggesting an increased cytotoxic T-cell presence. However, these findings should be interpreted with caution, as paired tumour tissue samples were only available for five participants in the exercise group and three in the control group [60]. In contrast, in a retrospective exploratory study [30], three sessions per week of supervised combined aerobic and resistance training for 12 weeks during NACT, no significant differences were observed in total tumour infiltrating lymphocytes between the exercise and control groups. Nevertheless, the mean reduction in tumour infiltrating lymphocyte levels from a biopsy to tumour was greater in the control group than in the intervention group [30]. Similarly, in patients with localised treatment-naïve prostate cancer scheduled for radical prostatectomy, a short-term, high-intensity interval training (HIIT) programme produced no significant differences in intra-tumoural CD3+ or CD8+ T-cell densities [61]. Notably, methodological limitations, including variability in exercise duration (2–8 weeks), and challenges related to tissue sampling and T-cell characterisation, may have masked a higher effect in this study [61].

In contrast, more data are available regarding systemic immune outcomes, though findings remain mixed. A recent meta-analysis in patients with breast cancer and survivors reported no significant effects of exercise interventions on circulating tumour-relevant immune subsets, such as CD4+, CD8+, and NK cells, likely owing to protocol variability, insufficient exercise dose, treatment effects, or methodological limitations [62]. Similarly, the aforementioned RCT by Ubink et al. [60] found no significant differences between the exercise and control groups in the proportions of major immune cell subsets in peripheral blood, including lymphocytes, monocytes, T cells, B cells, NK cells, and CD4+ and CD8+ T-cell subsets. However, a lower percentage of naïve CD8+ T cells was observed in the exercise group, suggesting a higher proportion of antigen-experienced CD8+ T cells (central memory, effector memory and effector memory cells that re-express CD45RA [EMRA]) compared with controls. Notably, the exercise group also showed increased expression of the degranulation marker CD107a on NK cells, and there was a trend toward enhanced in vitro tumour cell lysis [60]. These findings suggest that exercise during chemotherapy may enhance NK cell function, even in the absence of measurable changes in circulating immune cell proportions. Nevertheless, the study was limited by a small sample size and a heterogeneous breast cancer population in terms of the tumour subtype, so these results should be interpreted with caution and warrant confirmation in larger well-controlled trials.

The optimal exercise prescription for modulating immune outcomes remains unclear, and the impact of exercise may depend on training intensity, fitness level and age. In middle-aged and older women with BMI ≥ 25 kg/m^2^ and at high risk of breast cancer, neither moderate-intensity continuous training (MICT) nor HIIT improved NK cytotoxicity [63]. Nevertheless, those with lower baseline cardiorespiratory fitness experienced greater increases in NK cell activity, indicating that baseline fitness may influence training responsiveness [63]. Further analysis demonstrated that HIIT led to reductions in total CD4+ T cells, CD4+ naïve T cells, CD4+ recent thymic emigrants, and in the CD4:CD8 ratio, suggesting greater immunosenescence [64]. In contrast, MICT significantly increased total lymphocytes and CD8+ effector memory T cells, potentially supporting anti-tumour immune surveillance [64]. Moreover, post-intervention levels of total T cells, CD4+ naïve T cells, CD4+ central memory T cells and recent thymic emigrants were higher following MICT compared with HIIT, suggesting that moderate intensity may better preserve a youthful T-cell phenotype in ageing or overweight individuals. Interestingly, a near-significant inverse correlation was observed between improvements in VO_2_max and CD8+  EMRA T-cell numbers [64], a highly differentiated end-stage phenotype subset considered indicative of greater T-cell senescence. This may indicate that increases in aerobic fitness, typically greater with HIIT, may be required to reduce senescent T-cell populations. However, high-intensity training can also induce physiological stress that may blunt other immune benefits. For example, in HER2+ mouse models, only moderate-intensity aerobic exercise training (~ 60% VO_2_max) reduced tumour growth and enhanced CD8+ T-cell infiltration, whereas low and high intensities were ineffective or counterproductive [51]. Therefore, a combination of sustained MICT with periodic HIIT (~ 20% of total volume) may optimise both cardiorespiratory fitness [65] and immune outcomes. Similarly, in breast cancer survivors, an 8-week moderate-to-vigorous aerobic training programme delivered either partly supervised or remotely monitored reduced circulating CD4+ EMRA T cells in both groups and an improved CD4+ /CD8+ ratio in the partly supervised group, supporting the potential anti-immunosenescence effects of moderate-to-vigorous intensity aerobic training [66].

#### Treatment

Emerging evidence indicates that exercise may enhance the efficacy of cancer therapies, including radiotherapy, chemotherapy, immunotherapy, hormone therapy and targeted treatments, by modulating tumour biology, increasing blood perfusion, reducing hypoxia, improving drug delivery, and supporting antitumour immune responses.

##### Radiotherapy

Preclinical models have shown that aerobic training improves tumour vascularisation, increasing perfusion and oxygen delivery, thereby alleviating tumour hypoxia, a key factor limiting radiotherapy effectiveness [67–69]. For example, in lung cancer models, aerobic training increased tumour oxygenation and oxidative stress, sensitising tumours to radiation [70].

In prostate cancer models, exercise intensity and structure appear to be important. Forced treadmill running enhanced radiotherapy via NK cell activation and infiltration [71]. In contrast, voluntary wheel running had no effect, suggesting that structured higher-intensity exercise may be more effective than voluntary lower-intensity activity [68, 71]. In patients with breast cancer, resistance exercise training undertaken twice weekly for 12 weeks at moderate-to-high intensity during radiotherapy has been shown to reduce serum kynurenine and the kynurenine/tryptophan ratio, a marker of immune suppression, indicating potential systemic immunomodulatory benefits [72].

##### Chemotherapy

Preclinical studies in different cancer models have shown that aerobic training improves chemotherapy efficacy by enhancing tumour perfusion, reducing hypoxia, and promoting apoptosis [73–76]. In addition, as anti-tumour immune responses are critical to the efficacy of many chemotherapy agents [77], exercise may support chemotherapy outcomes through its effects on the immune system [47–49, 78]. Moreover, the interaction between the microbiome and the immune system may represent an additional pathway through which exercise, by modulating both, can enhance the effects of chemotherapy [79, 80].

In clinical studies in patients with breast cancer, increased lymphocyte infiltration during NACT correlates with a better treatment response [81, 82], suggesting that exercise-induced immune modulation may be beneficial during NACT treatment. Supporting this, preclinical models demonstrate that aerobic training can mobilise T cells that effectively infiltrate tumours and suppress growth [50, 52, 55, 57, 83–85]. However, human trials remain very limited. As discussed above, in patients with breast cancer undergoing NACT, Ubink et al. [60] showed a higher infiltration of CD56+ cells and a reduction in the CD4/CD8 ratio, while Guedes et al*.* [30] showed no differences in the number of TILs between groups. In pancreatic cancer, unsupervised combined aerobic training (brisk walking for 20–30 min at least 3 times/week) and resistance training (30 min, two times/week) for 17 weeks during NACT increased CD8+ T-cell infiltration compared with a control cohort [83]. Similarly, in oesophageal adenocarcinoma, a recent RCT showed that 16 weeks of low-to-moderate intensity combined aerobic and resistance training twice weekly supervised and three times weekly home-based exercise during NACT increased the number of CD8+ T cells and the levels of CD56+ in the tumours and induced favourable immune-related gene expression in the tumours [86]. Moreover, improvements in VO_2_max correlated with greater CD8+ cell infiltration, suggesting a link between increased aerobic capacity and anti-tumour immunity, although clinical outcomes were unchanged. These findings suggest that combined aerobic and resistance training at moderate intensity may enhance immune infiltration, which may be particularly important in patients undergoing NACT. Nevertheless, a systematic review has highlighted that only specific combinations of exercise type and intensity may elicit immune changes during chemotherapy or radiotherapy, with untailored protocols likely explaining null findings [87].

##### Immunotherapy

The immunomodulatory effects of exercise provide a strong rationale for combining with immunotherapy [48, 88]. Preclinical studies indicate that exercise enhances tumour control when combined with immune checkpoint inhibitors. In breast cancer models, moderate-to-vigorous aerobic training started post-implantation increased tumour perfusion, CD8+ T-cell infiltration and cytotoxic function, boosting the effects of immune checkpoint inhibitors [52, 55]. In a lung cancer model using NOD-SCIDγ mice (which lack T cells, B cells and mature NK cells), moderate-intensity combined aerobic and resistance training alone reduced tumour growth [89]. Although anti-programmed cell death protein 1 (PD-1) therapy provided no additional benefit on reducing tumour growth in this model, the combination increased tumour necrosis compared with non-intervention controls. In addition, exercise, alone or combined with anti-PD-1, reduced cell proliferation in the tumours compared with non-exercise. Moreover, exercise alone increased myeloid (primarily neutrophil) infiltration relative to the anti-PD-1-only group, suggesting that exercise may influence tumour progression via alternative immune mechanisms independent of the classical involvement of T lymphocytes or NK cells [89].

In contrast, in EO771 breast and B16-F10 melanoma models, voluntary exercise did not reduce tumour growth, and neither did anti-PD-1 or their combination, and in the breast cancer model, exercise alone reduced CD8+ T-cell infiltration [90]. The authors hypothesised that in aggressive tumours such as EO771, the combined energetic demands of tumour growth, immune activation and exercise may exceed physiological capacity, impairing anti-tumour T-cell responses. However, this contrasts with findings from Gomes-Santos et al. [52], where structured treadmill (forced) exercise increased CD8+ T-cell infiltration and cytotoxic function in EO771 tumours, suggesting that exercise modality and dosing may influence immune responses. Notably, combining exercise with anti-PD-1 restored CD8+ T-cell proportions in breast tumours but not in melanoma, suggesting tumour-specific effects. In both models, exercise and/or immunotherapy had no impact on NK cell infiltration [90].

Recent evidence highlights the role of the gut microbiome in exercise–immunotherapy synergy. In a BRAFV600E melanoma model, both pre- and post-implantation exercise, whether voluntary or forced treadmill running, significantly reduced tumour growth [91]. Exercise treatment was ineffective when CD8+ T cells were depleted, identifying these cells as key mediators of exercise-induced antitumour effects. Importantly, exercise-induced shifts in the gut microbiota were both necessary and sufficient for tumour suppression. Formate, a short-chain fatty acid (SCFA) produced by the microbiota, was identified as a central driver of this effect, enhancing tumour antigen-specific CD8+ T cell immunity, improving ICI response, and inhibiting tumour progression across multiple cancer models. Notably, high serum formate levels also correlated with longer progression-free survival in patients with melanoma, suggesting microbiota-derived formate can be both a mechanistic mediator and potential biomarker of exercise-enhanced immunotherapy.

Studies in humans are still very scarce. In a retrospective study, patients with unresectable hepatocellular carcinoma receiving lenvatinib (receptor tyrosine kinase inhibitor) and anti-PD-1 therapy who engaged in regular physical activity (> 150 min/week moderate or > 75 min/week vigorous intensity) had significantly improved survival and response outcomes [92]. Animal studies confirmed that the addition of exercise to lenvatinib plus PD-1 blockade suppressed tumour growth and extended survival, by reducing regulatory T-cell infiltration and downregulating checkpoint molecules [92]. Together, these findings underscore the potential of moderate-to-high aerobic training as an adjuvant to immunotherapy, while highlighting the importance of exercise mode, dose and tumour context in shaping immune responses and therapeutic outcomes.

##### Anti-HER2 Therapy

Although the effects of exercise on the efficacy of anti-HER2 therapies have not been studied, the immunomodulatory effects of exercise have potential synergy with anti-HER2 therapies, which rely partly on immune mechanisms such as antibody-dependent cytotoxicity, dendritic cell activation, and CD8+ T-cell-mediated responses [49, 93, 94]. Notably, treadmill running before combined doxorubicin and HER2-targeted treatment has been shown to protect against cardiac dysfunction in mice [95].

##### Hormone Therapy

Exercise may also improve the efficacy of hormone therapy. In mouse models, the combination of aerobic training with tamoxifen or letrozole reduced tumour volume more than either treatment alone, by modulating estrogen signaling, inflammation and apoptosis [96, 97]. A RCT in healthy women and patients with breast cancer receiving letrozole or tamoxifen examined the effects of 12 weeks of HIIT on circulating microRNAs (miRNAs) [98]. Compared with controls, patients with breast cancer had higher levels of oncogenic miRNAs and lower levels of tumour suppressor miRNAs. Exercise combined with hormone therapy led to a greater downregulation of oncogenic miRNAs and upregulation of tumour suppressor miRNAs compared with hormone therapy alone [98].

#### Summary of Tumour Subtype and Treatment

Taken together, emerging data suggest that the clinical outcomes of exercise in breast cancer are shaped by a complex interplay between tumour subtype and exercise modality, intensity and dose. Hormone receptor-positive tumours may be particularly influenced by interventions targeting metabolic and hormonal pathways, such as multimodal dietary and combined aerobic and resistance training approaches, while more immunogenic subtypes such as HER2+ and TNBC may be particularly responsive to a combination of moderate-intensity aerobic training combined with HIIT or moderate-to-high intensity aerobic training to support immunosurveillance, and resistance training to preserve muscle mass and myokine production [48, 57]. Overly intense protocols may induce physiological stress and blunt immune benefits in some individuals, especially those who are older or overweight. Nevertheless, the optimal exercise programme according to tumour subtype remains unclear.

In addition, exercise may act as a valuable adjunct to cancer therapies. These synergistic effects are mediated through multiple biological mechanisms, including improved tumour perfusion, modulation of immune cell infiltration and activation, alleviation of immunosuppression, regulation of hormonal pathways and modulation of the gut microbiome. Moderate-to-vigorous intensity aerobic training appears to be the most consistently effective approach across treatment modalities, especially when delivered at sufficient frequency and duration. Resistance training may provide additional benefits by preserving muscle mass and function, which not only improves overall metabolic and inflammatory profiles but may also expand the skeletal muscle reservoir for exercise-induced myokine release during both aerobic and resistance training. However, these proposed effects are largely supported by mechanistic and preclinical evidence and remain to be confirmed in clinical trials.

### Tailoring Exercise Based on Patient Characteristics

#### Genetics and Epigenetics

Inherited genetic variations, such as single nucleotide polymorphisms, contribute significantly to individual differences in exercise responsiveness and adaptation [13, 14]. For instance, *ACTN3* polymorphisms are associated with muscle power and sprint performance, while variants in insulin-like growth factor 1 (*IGF1*)*,* growth hormone receptor (*GHR),* AKT serine/threonine kinase 1 *(AKT1*) and myostatin (*MSTN*) genes have been linked to inter-individual variability in hypertrophic responses to resistance exercise [14, 99].

Muscle fibre type composition, which is approximately 45% heritable [100], also influences performance. A predominance of type I fibres favours endurance, while type II fibres benefit from high-intensity anaerobic activities [14]. Similarly, VO_2_max response to aerobic exercise shows a heritability of up to 50% after adjusting for age, sex and baseline fitness [12, 13].

Beyond general exercise responsiveness, emerging evidence indicates that physical activity may also influence breast cancer risk and progression in women with inherited cancer susceptibility. Observational studies have reported a reduced risk of breast cancer among *BRCA1/2* mutation carriers who engage in regular physical activity, particularly if initiated during adolescence or early adulthood [101, 102]. Preclinical studies further demonstrate that exercise upregulates BRCA1 messenger RNA in mammary tissue [102], and a pilot clinical trial reported improved antioxidant status and increased serum BRCA1 protein levels after a 6-week HIIT and resistance training programme in *BRCA* mutation carriers [103].

Epigenetic mechanisms, including DNA methylation, histone modifications and non-coding RNAs, mediate exercise adaptations and may partly explain variability in responsiveness [13, 14]. For instance, baseline differences in DNA methylation and transcriptomic profiles have been linked to variability in metabolic and cardiorespiratory responses to exercise, with high responders showing greater upregulation of mitochondrial-related genes in skeletal muscle in individuals with type 2 diabetes mellitus [13, 104]. Moreover, exercise directly induces epigenetic modifications [14]. For instance, acute exercise decreases DNA methylation in the promoter regions of key metabolic regulators, leading to enhanced gene expression [14]. Similarly, longer-term aerobic training promotes hypomethylation of genes involved in mitochondrial function, metabolism and insulin signalling [14, 105, 106], while resistance training upregulates genes related to extracellular matrix remodelling [106].

In breast cancer, exercise may counteract tumour-associated epigenetic changes. For example, in patients with breast cancer, aerobic training reduced promoter methylation of the tumour suppressor gene *L3MBTL1* in blood, and higher *L3MBTL1* expression, associated with reduced methylation, was correlated with improved survival [107, 108]. Furthermore, pre-diagnosis physical activity reduced breast cancer mortality only among women with tumours showing methylation of specific genes [107].

Exercise also modulates miRNA expression, upregulating tumour-suppressive and downregulating oncogenic miRNAs linked to poor prognosis in breast cancer [36]. These changes are associated with reduced tumour growth, proliferation, angiogenesis and epithelial-to-mesenchymal transition [36].

Incorporating genetic and epigenetic information into exercise assessment and prescription may enhance individual responsiveness and therapeutic benefits. While phenotypic assessments such as fitness and strength remain central to exercise planning, genetic insights could complement these assessments to inform longer-term training strategies. For example, resistance training may be favoured in individuals with lower hypertrophic or strength potential, and aerobic training in those with a lower genetic predisposition for VO_2_max, to help maximise their capacity. However, as both modalities are independently associated with improved breast cancer and OS [109], genetic insights may guide prioritisation rather than exclusion. In addition, exercise-induced epigenetic changes may provide a mechanistic basis for countering tumour progression, further supporting a precision approach to exercise oncology [36, 107, 108].

#### Gut Microbiome

The gut microbiome is a key regulator of host immunity, metabolism and inflammation, all of which are relevant to breast cancer progression and response to treatment [79, 110–112]. Exercise can modulate gut microbiota composition and function [113], providing a potential mechanism through which it may influence the tumour microenvironment and systemic health, potentially contributing to improved outcomes, though this remains to be confirmed in clinical studies.

In murine models, voluntary running increases alpha diversity and enhances the abundance of butyrate-producing taxa and SCFA production [49, 114–116], changes associated with improved immune function and enhanced responses to cancer therapies [79, 110, 111, 117–119]. Notably, this relationship appears bidirectional, as the gut microbiota also influences skeletal muscle mass and function through the production of SCFAs and the absorption of nutrients, highlighting the interconnected nature of the muscle–gut axis [113, 120].

In humans, cross-sectional studies in athletes show greater microbial diversity and a higher abundance of butyrate-producing bacteria compared with sedentary individuals [80, 121–123]. Longitudinal studies in sedentary individuals have reported similar shifts after training, including increased *Akkermansia* sp. abundance [80, 124], increased microbial diversity [125], higher SCFA levels [125, 126], and reduced systemic and gut inflammation [127].

Exercise type, intensity and duration appear to influence gut microbiome composition [114]. Studies show that combined aerobic and resistance training may induce greater changes in microbial diversity than aerobic training alone [114], although aerobic exercise more consistently affects specific taxa [114]. Moderate-to-high intensity exercise performed 3–5 times per week for 30–90 min over at least 8 weeks seems most effective in enhancing alpha diversity [114]. However, exceeding this range may transiently increase gut permeability and inflammation, suggesting an inverted-U relationship [114, 116].

Interestingly, fitness status may influence the microbiome more than training load alone [116]. Higher cardiorespiratory fitness, rather than just the volume or intensity of exercise performed, is associated with greater microbial diversity and abundance of health-promoting taxa [80, 116, 128]. This suggests that sustained fitness, influenced by both consistent training and genetic predisposition, creates a more stable and resilient microbiome environment. In contrast, temporary increases in the exercise training load may only induce transient changes in gut microbiota, which often reverse after cessation of training [114, 116, 120, 126]. This has important implications for cancer care, where ongoing fitness maintenance rather than episodic training might be key to modulating the gut microbiome in ways that may, in turn, support immunity, treatment tolerance and long-term survivorship.

Data in patients with cancer remain very limited [129]. In breast cancer survivors, higher fitness levels correlate with greater microbial diversity and an abundance of beneficial taxa [130, 131], and similar associations have been reported in colorectal cancer survivors [129, 132]. In a study of breast cancer survivors who had received chemotherapy, a 12-week supervised combined aerobic and resistance training programme showed no significant changes in alpha diversity [133]. However, exercise induced changes in specific bacterial taxa, and faecal transplants of post-exercise samples from a participant that demonstrated a favourable microbial response to exercise reduced tumour growth in mice compared to pre-exercise transplants. This suggests that exercise-induced changes in the gut microbiome may mediate the anti-tumour effects of exercise [133].

Importantly, interindividual differences in the gut microbiome may influence responsiveness to exercise. Baseline microbiome profiles have been shown to predict metabolic responses to exercise [134], and variability in microbiome adaptations has been observed in cancer survivors, potentially influenced by factors such as BMI and metabolic status [133]. Future research is needed to clarify how exercise prescription components affect the microbiome in oncology settings, and how interindividual differences in the microbiome composition may influence responsiveness to exercise [129, 135]. This will support the development of tailored exercise strategies that promote a more diverse and resilient gut microbiota and optimise clinical outcomes in patients with cancer.

#### Chronic Inflammation

Chronic inflammation is a major driver of tumour progression and carcinogenesis, further exacerbated by treatments such as radiotherapy and chemotherapy [136]. Exercise has been shown to modulate systemic inflammation in patients with breast cancer and survivors, though effects vary across biomarkers, intervention types and study design. A recent Bayesian network meta-analysis of RCTs in patients with cancer demonstrated that exercise interventions significantly reduced tumour necrosis factor-α (TNF-α) and C-reactive protein (CRP) levels, with high-intensity aerobic training combined with resistance training providing the greatest overall benefits [136]. In breast cancer-specific analyses, exercise reduced CRP and interleukin (IL)-6 levels post-treatment, although effects on TNF-α were inconsistent [136].

Additional meta-analyses support these findings. Abbasi et al. [137] found that in patients with breast cancer, exercise significantly reduces CRP levels, especially following long-term combined aerobic and resistance training, although no significant effects were seen for IL-6, TNF-α or other cytokines compared to controls. Meneses-Echávez et al. [138] and Bettariga et al. [139] observed reductions in IL-6, TNF-α, and IL-8 following structured exercise interventions (particularly combined aerobic and resistance training) in breast cancer survivors, while findings for CRP and IL-10 were inconsistent across studies. Overall, combined aerobic and resistance training appears most effective in reducing proinflammatory cytokines, though effects vary by biomarker and study design.

Importantly, total weekly exercise volume and intervention duration may influence the levels of different proinflammatory cytokines. Larger reductions in TNF-α were seen with higher weekly exercise volumes [136]. Interventions > 12 weeks were more effective at lowering IL-6, while shorter interventions (≤ 12 weeks) showed greater reductions in CRP and IL-10 [140]. Similarly, longer sessions (> 45 min) and programmes over 11 weeks improved IL-6 [138], while IL-8 reductions were evident in programmes under 16 weeks [139]. Thus, it appears that the anti-inflammatory effects of exercise may vary by biomarker, with some (e.g. IL-6, TNF-α) requiring sustained interventions, while others (e.g. CRP, IL-10, IL-8) may respond earlier.

Physiological adaptation, such as strength gains or fat mass reduction, may also mediate inflammatory outcomes. For example, in a secondary analysis of three resistance training trials in breast cancer survivors, reductions in CRP were more pronounced among women who gained strength, and both CRP and leptin were reduced among those who lost at least 2 kg of body weight [141]. Improvements in strength and reductions in fat mass may influence inflammation through distinct biological mechanisms, via myokine release from skeletal muscle and decreased proinflammatory cytokine secretion from adipose tissue, respectively. Together, these findings support the role of structured exercise programmes, particularly those that combine aerobic and resistance training, as strategies to mitigate low-grade chronic inflammation and potentially influence breast cancer prognosis. However, the optimal intensity, volume, frequency and duration remain to be fully determined.

#### Obesity and Metabolic Dysfunction

Obesity is a well-established risk factor for worse outcomes in breast cancer survivors [142–144]. It contributes to tumour progression through altered glucose metabolism, elevated circulating estrogens, insulin resistance and chronic low-grade inflammation [143, 145, 146]. Similarly, metabolic syndrome, a cluster of dysfunctions including central obesity, dyslipidaemia, hypertension and insulin resistance, is associated with poorer breast cancer outcomes [147].

Exercise is an effective strategy to improve body composition and mitigate metabolic dysfunction in both patients with breast cancer and survivors. In patients undergoing chemotherapy, a meta-analysis by Li et al. [148] found that exercise reduced body weight, BMI, and body fat percentage, and improved muscular strength. These findings are consistent with those from other meta-analyses in patients with breast cancer and survivors, which showed that combined aerobic and resistance training significantly improved body weight, BMI, body fat percentage, fat mass, waist circumference and fat-free mass [149, 150].

When comparing exercise types, several studies suggest that combined aerobic and resistance training is the most effective approach for improving body composition. In breast cancer survivors, combined aerobic and resistance training is more effective than aerobic or resistance training alone for improving both BMI and body fat percentage, with benefits greatest in programmes exceeding 12 weeks and 150 min per week [145]. Another meta-analysis similarly found that combined aerobic and resistance training was superior in overweight or obese patients with breast cancer and survivors for improving BMI, fat mass, body fat percentage, hip circumference and fat-free mass [150]. Specific aerobic exercise training prescriptions such as HIIT have also been compared to resistance exercise training. In a recent RCT in breast cancer survivors, both interventions improved body composition, but resistance training was more effective at increasing absolute lean mass and strength, whereas HIIT achieved slightly greater reductions in fat mass [151]. In overweight or obese breast cancer survivors, while both HIIT and MICT reduced body mass and fat mass, HIIT produced slightly greater improvements in both measures [152]. High-intensity interval training may therefore be a useful tool, particularly when the goal is fat loss, while resistance training remains essential for preserving or increasing muscle mass.

To achieve meaningful weight loss, exercise should be combined with dietary management. Caloric restriction, alone or when combined with aerobic and resistance training or physical activity, is most effective for reducing fat mass, body weight, and BMI in patients with breast cancer during and following treatment [153]. However, caloric restriction can also result in the loss of lean tissue. Resistance training therefore remains essential to prevent lean mass loss commonly associated with caloric restriction [153]. This is particularly relevant given that low muscle mass has been consistently associated with poorer survival outcomes in patients with breast cancer [154–156].

Exercise can also mitigate metabolic dysfunction. A RCT in overweight and obese breast cancer survivors (waist circumference > 88 cm) demonstrated that a 16-week programme of combined moderate-to-vigorous aerobic and resistance training significantly reduced the prevalence of metabolic syndrome, from 77% at baseline to 15% post-intervention, compared with 80% in the usual care group [42]. The intervention also improved body composition as well as insulin sensitivity, lipid profiles and inflammatory markers [42]. These benefits are supported by mechanistic evidence showing that aerobic exercise enhances mitochondrial biogenesis, glucose transporter type 4 (GLUT-4) translocation and fatty acid oxidation, while resistance exercise contributes to increased glucose uptake and insulin sensitivity by stimulating muscle growth and glycogen storage [145, 157].

Several systematic reviews and meta-analyses consistently support the metabolic benefits of exercise in breast cancer populations, particularly with combined aerobic and resistance training performed for ≥ 12 weeks. Improvements in fasting glucose [149], fasting insulin [149, 158, 159] and insulin resistance [138, 149, 158] have been reported across multiple studies. Exercise also improves lipid profiles, with increases in high-density lipoprotein-cholesterol, and reductions in triglycerides, total cholesterol and low-density lipoprotein-cholesterol observed in both healthy and overweight/obese breast cancer populations [149, 150, 158]. Additionally, combined aerobic and resistance training has been associated with reductions in blood pressure [149] and favourable changes in adipokines [158]. However, findings on insulin-like growth factors (IGFs) and IGF binding proteins are mixed, with some reviews showing no significant changes in IGF-I or IGF binding proteins [158, 159], while others report reductions in IGF-I/II and increases in IGF binding proteins 1/3 [138, 140].

When comparing exercise types, combined aerobic and resistance training appear most effective for improving glycaemic control [145]. Therefore, combined moderate-to-vigorous or high-intensity combined aerobic and resistance training programmes should be implemented to address obesity and metabolic dysfunction in patients with breast cancer and survivors.

#### Summary of Patient Characteristics

In summary, biological features such as genetics, epigenetic patterns and gut microbiota composition not only influence individual responses to exercise, but gene expression and microbiome composition can also be modified through exercise. However, we are not yet at a point where these factors can guide specific exercise prescriptions in clinical practice. While exercise can already be used to target modifiable conditions such as chronic inflammation, obesity and metabolic dysfunction, using biological profiles to develop precision exercise strategies remains a promising but still exploratory area. Defining which exercise strategies are most effective for which biological profiles will be a key step in advancing precision exercise approaches in breast cancer care.

## Safety and Clinical Implementation Considerations

Given the translational focus of precision exercise, clinical implementation should account for treatment-related toxicities, disease-specific factors, and comorbidities that may influence exercise tolerance and safety. Current oncology exercise recommendations suggest that exercise prescription should be adapted according to the predominant symptom or risk profile rather than applied uniformly [11].

For example, in patients with anaemia, shorter and more frequent aerobic bouts may be preferable, with intensity guided by perceived exertion or breathlessness rather than heart rate, and with closer supervision when symptoms are more pronounced [160]. In patients with bone metastases, exercise should not necessarily be avoided, but prescription should be individualised according to lesion location and presentation, with emphasis on postural alignment, controlled movement, proper technique, gradual progression and avoidance of excessive loading of fragile skeletal sites [161]. Similarly, in patients with or at risk of lymphedema, progressive resistance training appears feasible and may be beneficial [162], provided symptoms are closely monitored and supportive strategies, including compression garments when appropriate, are individualised.

Treatment-related toxicities such as neutropenia, fatigue, peripheral neuropathy and cardiotoxicity (e.g. associated with anthracyclines or anti-HER2 therapies) may require temporary modifications in exercise mode, intensity, supervision and training environment [11]. For example, higher-intensity protocols such as HIIT or high-load resistance training may need to be modified, dosage lowered or completely avoided, in patients with significant fatigue, cardiopulmonary limitations or uncontrolled symptoms, especially during active treatment.

From a practical perspective, exercise intensity and progression should be guided using symptom-based approaches rather than fixed prescriptions. Tools such as rating of perceived exertion or subjective scales may be preferable to heart rate-based methods, particularly in patients undergoing active treatment. Exercise programmes should follow a gradual and flexible progression model, adapting to day-to-day fluctuations in symptoms and treatment-related side effects.

Clinical screening should be conducted in all patients prior to exercise participation to identify comorbidities, symptoms and potential risks. Additional medical clearance pathways may be required for patients with significant comorbidities, severe treatment-related toxicities or high-risk conditions such as bone metastases or cardiotoxicity risk. Ongoing clinical monitoring, including regular assessment of symptoms, functional status and treatment tolerance, is essential to ensure that exercise remains both safe and appropriate throughout the cancer care continuum.

## Future Directions: Applying Data Integration and Digital Tools

Looking forward, the application of precision exercise will increasingly rely on the integration and processing of multi-dimensional data derived from clinical, biological and behavioural sources (Fig. 2). Emerging data-driven tools such as AI, machine learning, wearable technologies and multi-omics profiling offer new opportunities for precision exercise interventions. Artificial intelligence is currently being applied in exercise and health programmes for multiple functions, including automated messaging, gesture recognition, predictive modelling, data collection and guided training, targeting outcomes such as pain, performance and event prediction [163]. Furthermore, advances in omics technologies and wearable sensors offer a deeper insight into the biological variability underlying exercise responses. For example, the Molecular Transducers of Physical Activity Consortium (MoTrPAC), a large-scale National Institutes of Health-funded initiative, is generating multi-omics datasets from humans and animal models to map the molecular effects of acute and chronic aerobic and resistance exercise training [164]. These data will help to identify the biological pathways through which exercise influences health and disease, and ultimately the guidance of more personalised and effective training strategies [164].

Similarly, the Digital Twins project led by Spain’s National Cancer Research Centre (CNIO) [165, 166] is generating individualised computational models (digital replicas) of women with advanced breast, lung or colorectal cancer. These virtual models integrate clinical data, multi-omics profiles (genomics, proteomics, metabolomics, microbiome), lifestyle and behavioural patterns (e.g. physical activity, diet, mobile phone usage) and psychosocial factors (e.g. quality of life, emotional well-being). Data are collected through wearables and smartphone apps, and synthesised using AI algorithms to predict disease progression, evaluate treatment efficacy and inform lifestyle interventions. Although not exclusively focused on exercise, this project has profound implications for precision exercise by uncovering how physiological and behavioural factors influence cancer outcomes.

Recent studies have begun to implement personalised exercise interventions tailored to the individual needs, treatment phase and physiological status of patients with cancer. For example, Ryu et al. [167] designed an ongoing 12-month home-based programme for patients with breast cancer tailored to surgery type, treatment phase and physical function, aiming to improve recovery, cardiometabolic health and quality of life. Outcomes include insulin, inflammatory markers, the breast tumour marker carcinoembryonic antigen, body composition, patient-reported outcomes and gut microbiome composition. Building on this direction, Gao et al. [168] proposed a protocol to develop a machine learning-based system for generating precision exercise prescriptions aimed at cardiovascular disease prevention in cancer survivors and individuals with stage I cancer, using wearables-derived data and machine learning algorithms to guide ongoing adjustments.

Together, these initiatives demonstrate the potential of digital tools and biological data to drive biomarker-informed precision exercise strategies in oncology (Figs. 1 and 2). Nevertheless, while digital health tools, multi‑omics profiling and AI offer promising avenues for biomarker‑informed precision exercise, their clinical implementation in oncology remains limited. The integration of high‑dimensional biological data (e.g. genomics, metabolomics, microbiome, immune profiling) raises important issues related to data governance, including privacy protection, informed consent, and data ownership particularly when these data are combined with continuous information from wearables or digital platforms that enable long‑term monitoring of patients’ real‑world behaviours.

From a methodological perspective, algorithm‑based decision tools may be vulnerable to bias and limited generalisability if they are trained on small, unrepresentative or highly adherent trial populations, a common limitation in exercise oncology research. In parallel, limited interpretability of complex models can hinder clinical adoption when recommendations cannot be clearly explained in terms of underlying biological or clinical factors.

In addition, the implementation of digitally enabled precision exercise approaches is constrained by cost and infrastructure requirements, including access to omics testing, data storage and analysis capacity, and specialised multidisciplinary expertise. Finally, in the absence of validated and clinically actionable biomarkers, there is a risk of over‑personalisation, whereby increasingly granular recommendations are generated without robust evidence of benefit on oncological outcomes. Together, these considerations underscore the need for cautious interpretation, rigorous validation and implementation science before widespread adoption.

## Conclusions

Current evidence supports the beneficial role of structured exercise in improving a range of patient‑reported, functional and biological outcomes in breast cancer. However, evidence linking exercise to survival outcomes and tumour response is based mainly on observational data and exploratory analyses from interventional trials, and therefore, cannot establish causality [24, 25, 27]. Combined aerobic and resistance training performed at moderate-to-high intensity appears to be the most effective in improving body composition, reducing systemic inflammation, enhancing myokine release, modulating the gut microbiome, and sustaining immune function and immune surveillance, which are particularly relevant during active treatment. Incorporating some aerobic training sessions as HIIT may provide additional metabolic, hormonal and immune benefits; however, in older or unfit patients, a more gradual and progressive approach is recommended to avoid excessive physiological stress that could counteract the immune benefits.

To support the potential clinical application of precision exercise in breast cancer, Tables 1 and 2 show a framework that maps tumour characteristics, treatment modalities and patient characteristics to relevant biological targets, clinical outcomes and suggested exercise strategies. Table 1 focuses on tumour and treatment-specific factors, highlighting how exercise may influence the hormonal milieu, the tumour microenvironment, vascular function, and immune infiltration and function. For patient-related characteristics, Table 2 shows how exercise may modulate biological mechanisms that can affect clinical outcomes, such as gene expression, microbiome composition and function, body composition, insulin sensitivity and systemic inflammation. In most cases, a combination of aerobic and resistance training is suggested based on current evidence. However, specific FITT (frequency, intensity, time, type) parameters remain to be optimised and are likely to vary depending on individual characteristics.

**Table 1:** Overview of exercise recommendations based on tumour subtype and treatment: a conceptual framework. The exercise strategies presented are conceptual and hypothesis generating, based on a synthesis of clinical and preclinical mechanistic evidence supporting the proposed biological mechanisms. These strategies are intended to illustrate potential biological rationales for precision exercise prescriptions and should not be interpreted as formal clinical guidelines

Characteristic	Biological target	Clinical objective	Proposed exercise prescription	Evidence strength	Key refs
HR+ /HER2**-** tumours	↓ Estrogens ↓ Chronic inflammation ↓ Adiposity	Support hormonal therapy Reduce recurrence Increase long-term survival	AE (moderate-to-high intensity) + RE + dietary intervention	Moderate (clinical evidence for metabolic and hormonal effects)	[39, 43]
HER2+ tumours	↑ Immunosurveillance ↑ Naïve T cells ↓ Dysfunctional T cells ↑ CD8+ T cells and NK cell infiltration ↓ TME immunosuppression ↑ Myokines release from muscle (IL-6, IL-7, IL-15) ↑ Gut microbiome diversity and SCFA production	Increase treatment efficacy Reduce recurrence Increase long-term survival	AE (moderate-to-high intensity) + RE in older populations AE: moderate-to-high intensity + RE with potential inclusion of HIIT in younger populations (e.g. 80% moderate-to-high intensity + 20% HIIT)	Low-to-moderate (mainly preclinical; limited and exploratory clinical evidence)	[60, 64]
TNBC	↑ Immunosurveillance ↑ Naïve T cells ↓ Dysfunctional T cells ↑ CD8+ T cells and NK cell infiltration ↓TME immunosuppression ↑ Myokines release from muscle (IL-6, IL-7, IL-15) ↑ Gut microbiome diversity and SCFA production	Increase treatment efficacy Reduce recurrence Increase long-term survival	AE (moderate-to-high intensity) + RE in older populations AE: moderate-to-high intensity + RE with potential inclusion of HIIT in younger populations (e.g. 80% moderate-to-high intensity + 20% HIIT)	Low-to-moderate (mainly preclinical; limited and exploratory clinical evidence)	[60, 64]
Chemotherapy	↑ Blood perfusion ↓ Tumour hypoxia ↑ Immunosurveillance ↑ CD8+ T cells and NK cells infiltration ↓ Immunosuppression ↑ Gut microbiome diversity and SCFA production	Increase CT efficacy Reduce recurrence Increase long-term survival	Supervised AE + RE, moderate intensity, 2–3 × /week	Low to moderate (mainly preclinical; limited and exploratory clinical evidence)	[60, 73, 75]
Radiotherapy	↑ Blood perfusion ↓ Tumour hypoxia ↑ CD8+ T cells and NK cells infiltration	Increase RT efficacy Reduce recurrence Increase long-term survival	Supervised AE (moderate-to-high intensity)	Low (mainly preclinical, very limited clinical evidence)	[69, 72]
Immunotherapy	↑ CD8+ T cells infiltration ↓ Immunosuppression ↑ Gut microbiome diversity and SCFA production	Increase IT efficacy Reduce recurrence Increase lon- term survival	Supervised AE (moderate-to-high intensity)	Low (mainly preclinical, very limited clinical evidence)	[51, 55, 92]

*AE* aerobic exercise, *CT* chemotherapy, *HER2+* human epidermal growth factor receptor-positive, *HER2-* human epidermal growth factor receptor-negative, *HIIT* high-intensity interval training, *HR* hormone receptor, *IL* interleukin, *IT* immunotherapy, *NK* natural killer, *RE* resistance exercise, *refs* references, *RT* radiotherapy, *SCFA* short-chain fatty acids, *TME* tumour microenvironment, *TNBC* triple-negative breast cancer, ↓ decreased, ↑ increased

**Table 2:** Overview of exercise recommendations based on patient characteristics: a conceptual framework. The exercise strategies presented are conceptual and hypothesis generating, based on a synthesis of clinical and preclinical mechanistic evidence supporting the proposed biological mechanisms. These strategies are intended to illustrate potential biological rationales for precision exercise prescriptions and should not be interpreted as formal clinical guidelines

Characteristic	Biological target	Clinical objective	Proposed exercise prescription	Evidence strength	Key refs
Genetics and epigenetics	↑ VO_2_max	Increase long-term survival	Prioritise AE: moderate-to-high intensity over RE with potential inclusion of HIIT	Moderate (non-oncologic clinical evidence; limited cancer-specific data)	[12, 13]
Genetics and epigenetics	↑ Hypertrophy and muscle strength	Increase long-term survival	Prioritise RE over AE	Moderate (non-oncologic clinical evidence; limited cancer-specific data)	[14, 100]
Genetics and epigenetics	↑ Tumour suppressors’ expression ↓ Oncogenes’ expression	Reduce progression and recurrence Increase long-term survival	AE (moderate intensity or HIIT) + RE	Low (mainly preclinical, very limited clinical evidence)	[103, 107]
Gut microbiome	↑ Diversity ↑ SCFA production ↑ *Akkermansia* sp. ↑ Muscle mass and function ↑ Cardiorespiratory fitness ↑ Antitumoural immunity	Increase treatment efficacy Reduce progression and recurrence Increase long-term survival	AE (moderate-to-high intensity) + RE, 3–5 × /week, 30–90 min/session, long duration	Low (mainly preclinical, and non-cancer populations)	[113, 115, 116, 130, 133]
Chronic inflammation	↓ TNF-α, CRP, IL-6 ↓ Adiposity ↓ VAT ↑ Muscle strength	Reduce progression and recurrence Increase long-term survival	AE + RE	Moderate-high (meta-analyses in cancer populations)	[136, 137]
Obesity, metabolic syndrome	↓ Adiposity ↓ Waist circumference, ↓ VAT ↑ Lean mass ↑ Insulin sensitivity ↓ Dyslipidaemia ↓ Hypertension	Reduce progression and recurrence Increase long-term survival	AE (moderate-high + HIIT) + RE, ≥ 150 min/week + dietary intervention	Moderate-high (meta-analyses in cancer populations)	[148, 149, 153]

*AE* aerobic exercise, *CRP* C-reactive protein, *HIIT* high-intensity interval training, *IL* interleukin, *RE* resistance exercise, *refs* references, *SCFA* short-chain fatty acids, *TNF-α* tumour necrosis factor-α, *VAT* visceral adipose tissue, *VO*_*2*_*max* maximal oxygen consumption, ↓ decreased, ↑ increased

Precision exercise should involve the dynamic adjustment of the training variables and the application of the principles of training to match individual patient needs. As the field advances, translating insights from omics data, clinical profiles and digital monitoring into precision exercise prescriptions based on host, tumour and treatment characteristics represents a promising but complex challenge.

Most biological insights, including those from genomics, epigenetics and the microbiome, remain hypothesis generating and are not yet clinically actionable. In addition, although inter-individual variability in physiological and patient-reported responses to exercise is well established, it remains unclear whether this variability translates into differential effects on tumour response, recurrence or survival. Further investigation is also required to clarify immune responses to different exercise modalities across tumour subtypes and treatment contexts.

Consequently, we remain far from being able to routinely prescribe precision exercise programmes that integrate all these variables (Table 3). In this context, precision exercise in breast cancer should currently be viewed as a conceptual and hypothesis-generating framework that requires validation through adequately powered trials and mechanistic studies before clinical implementation. Furthermore, while tumour control is an important goal, precision exercise prescriptions must also prioritise other clinical needs, such as managing cardiovascular disease, preserving muscle mass in the context of frailty or mitigating cachexia, depending on the patient’s overall condition. Several key questions remain:

- How should exercise prescriptions be adapted according to host characteristics (e.g. genetics, metabolic status), tumour biology (e.g. HR+, HER2+, TNBC) or treatment modality (e.g. chemotherapy, immunotherapy)?
- What is the optimal exercise type and dose for improving clinical outcomes and for inducing meaningful biological changes in different patient phenotypes (e.g. obese, older, immunocompromised)?
- Which biomarkers (e.g. genomic, epigenetic, immune, microbial) best predict exercise responsiveness?
- How long-lasting are exercise-induced changes (e.g. in immune function, epigenetics or microbiome composition)?
- Will digital health tools and multi-omics approaches be able to be effectively integrated into routine care to guide precision exercise prescriptions?

**Table 3:** Precision exercise: are we there yet? A distinction between clinically actionable individualisation and emerging precision exercise interventions

*Clinically actionable now* Exercise can already be tailored in the clinical practice using established principles (FITT), based on:	*Emerging/experimental* Approaches aiming to guide exercise prescription using biological data are under investigation but are not yet clinically actionable:
Treatment context and treatment-related toxicities (e.g. fatigue, neuropathy, cardiotoxicity) Functional status and fitness level Body composition and metabolic health (e.g. obesity, sarcopenia, insulin resistance) Comorbidities (e.g. low bone mineral density, osteoarthritis, sarcopenia) Patient preferences and tolerability	Tumour and host genomics, proteomics, metabolomics Treatment efficacy Gut microbiome profiles Immune phenotyping and inflammatory markers Multi-omics integration and AI-driven models
This approach represents clinical individualisation, which is feasible, evidence informed, and essential to optimise safety, adherence and overall health outcomes	These approaches have expanded our understanding of inter-individual variability; however, they remain largely hypothesis generating. Key limitations include the lack of validated biomarkers, insufficient evidence linking biological profiles to differential exercise responses, and the complexity and cost of clinical implementation

Precision exercise aims to tailor exercise prescriptions based on patient, tumour and treatment characteristics. However, an important distinction should be made between clinically actionable individualisation and biomarker-informed precision exercise, which remains largely exploratory. In this context, precision exercise should be viewed not as an immediate clinical reality, but as a conceptual framework to guide the development of more targeted and effective interventions

*AI* artificial intelligence, *FITT* frequency, intensity, time, type

Future studies should include adequately powered RCTs comparing exercise types and doses and designed to evaluate effects across tumour subtypes, metabolic/inflammatory status and treatment regimes, while incorporating molecular and immune profiling to better understand underlying mechanisms and refine precision. Integration of wearable technologies and AI-driven decision support tools may facilitate real-time exercise programming, allowing adjustments based on patient progress and physiological feedback.

Beyond biological and technological aspects, implementation must also consider individual-level factors not covered in this review, such as comorbidities, treatment side effects, psychological state and personal preferences, which significantly influence participation and outcomes. Ultimately, precision exercise in breast cancer needs to move beyond proving feasibility and focus on personalised implementation, aligning exercise prescriptions with individual patient and disease characteristics. Integrating biological data, digital tools and clinical insight will be essential to advance this field.
